# Beyond histology: A tissue algorithm predictive of post-surgical recurrence in hepatocellular carcinomas, including *TERT* promoter mutation

**DOI:** 10.1007/s00428-024-03791-y

**Published:** 2024-05-18

**Authors:** Francesco Vasuri, Stefano Chillotti, Thais Maloberti, Elisa Albertini, Giuliana Germinario, Matteo Cescon, Matteo Ravaioli, Dario de Biase, Antonia D’Errico

**Affiliations:** 1 Pathology Unit, IRCCS Azienda Ospedaliero-Universitaria Di Bologna, Bologna, Italy; 2 School of Anatomic Pathology, Department of Biomedical and Neuromotor Sciences, University of Bologna, Bologna, Italy; 3 Solid Tumor Molecular Pathology Laboratory, IRCCS Azienda Ospedaliero-Universitaria Di Bologna, Bologna, Italy; 4 Department of Pharmacy and Biotechnology (FaBit), University of Bologna, Bologna, Italy; 5 Hepato-Biliary and Transplant Unit, IRCCS Azienda Ospedaliero-Universitaria Di Bologna, Bologna, Italy; 6 Department of Medical and Surgical Sciences (DIMEC), University of Bologna, Bologna, Italy

**Keywords:** Hepatocellular carcinoma, Histopathology, Next-generation sequencing, *TERT* promoter, Tumor recurrence

## Abstract

Surgical resection for hepatocellular carcinoma (HCC) is burdened with a high recurrence rate and a lack of reliable prognostic factors. The aim of this study was to integrate the HCC pathological features with gene mutations to improve the prognostic role of pathological analysis. This is a monocentric prospective study, including 67 patients resected for HCC. All clinical data and histological features were collected, including tumor grade, architecture, margins, microvascular invasion, and microscopic portal vascular invasion (MPVI). Next-generation sequencing (NGS) was performed using a laboratory-developed multi-gene panel, allowing to amplify 330 amplicons (21.77 kb), covering the relevant targets for solid tumor analysis. The most represented mutations were *TERT* promoter (*n* = 41, 61.2%), *TP53* (*n* = 18, 26.9%) and *CTNNB1* (*n* = 17, 25.4%). At follow-up, 13 (19.4%) patients experienced HCC recurrence: at multivariate analysis, tumor dimensions (*p* = 0.040), MPVI (*p* = 0.010), and *TERT* mutation (*p* = 0.034) correlated with recurrence. Dimensions ≥ 4.5 cm (very close to AJCC stage pT3; 9 recurrences, *p* = 0.041, odd-ratio = 3.7), MPVI (9 recurrences, *p* = 0.062, OR = 3.3), and *TERT* (11 recurrences, *p* = 0.049, OR = 4.4) correlated with disease-free survival also at univariate analysis. The concomitant occurrence of these three variables was present in 7 cases, among which 5 recurred (*p* = 0.002, OR = 15.94). In conclusion, NGS analysis in resected HCC could not only be used for future therapies but should be integrated with histopathology to predict the risk of tumor recurrence after surgical resection: *TERT* mutation is among the strongest predictors of tumor recurrence, together with tumor stage (dimensions) and the occurrence of MPVI, which should always be reported separately from the classic MVI.

**Supplementary Information:**

The online version contains supplementary material available at 10.1007/s00428-024-03791-y.

## Introduction

Surgical resection is one of the treatments of choice for hepatocellular carcinoma (HCC) in not cirrhotic livers, as well as in very early and early-stage HCCs in cirrhotic livers with good function [1]. According to the European Association for the Study of the Liver (EASL), the recurrence rate of HCC after any therapy—including de novo new HCCs—is still very high (up to 70%), impacting long-term patient prognosis [1, 2]. Compared to other therapeutic options, surgical resection has the advantage of giving more predictive information, due to the availability of tumor tissue and histopathological features associated with HCC aggressiveness, such as grade, architecture, and microvascular invasion (MVI) [3, 4]. Recently, the presence of MVI in the portal veins (microscopic portal vascular invasion, MPVI) was associated with a worse prognosis, in terms of direct correlation with serum alpha-fetoprotein and post-surgical tumor recurrence, compared to “simple” MVI [5]. This demonstrates how histopathological analysis can still be contributory, albeit its role in the prediction of HCC recurrence is still underrated since no standard pathological algorithms exist.

The availability of tumor tissue has the advantage of allowing molecular analyses as well: in the last years, many approaches have been proposed, from microRNAs [6, 7] to gene hypermethylations [8, 9] and mutational profile, especially mutations in the promoter telomerase reverse transcriptase gene (*TERT*) [10, 11]. It is established that HCC, despite the wide clinical and morphological heterogeneity, is typically not burdened by a high number of mutations, the most common driver mutations being in the *TP53*, *CTNNB1*, and *TERT*-promoter genes [10, 12]. The β-catenin and p53 pathways have been proposed for a molecular classification of HCC [10, 13], but the lack of strong evidence and clinical applications left these findings in the theory. The *CTNNB1* gene (encoding for β-catenin) is mutated in approximately one-third of HCC, described as low-grade trabecular-acinar tumors by the original work by Calderaro et al. [13] The frequency of *TP53* mutations in HCC is variable according to the series considered in the literature, from 15 to 40%: *TP53* mutation characterizes high-grade, more aggressive HCC, and it was correlated to aflatoxin exposure and HBV infection [10, 13]. *TERT* promoter is far less studied in liver cancers, also because this promoter region is not covered by the exome sequencing methods usually used. However, more than half of HCCs show *TERT* promoter mutation, and some preliminary studies showed a promising correlation between *TERT* mutation and worse prognosis in HCC patients [11, 14].

The state-of-the-art routine management of resected HCC patients does not include a tissue algorithm able to combine the usual histopathological variables with molecular biology data (as it is in other human cancers), and the fact that no correlations seem to exist between HCC morphology and mutational status discourages further the research of a more rational approach [15]. The aim of the present study was to integrate the HCC pathological features with gene mutations to find a prognostic tissue algorithm in resected HCC patients, improving the prognostic role of pathological analysis.

## Materials and methods

### Disclosure of ethical statements

Approval of the research protocol: Approved by the Ethical Committee of the Area Vasta Emilia-Romagna (AVEC), protocol Nr. *HCCNGS_2021*.

Informed Consent: informed consent was obtained by all enrolled patients.

Registry and the Registration No. of the study/trial:

Animal Studies: N/A.

Research involving recombinant DNA: N/A.

### Patient enrollment

This is a monocentric prospective study, in conformity with the ethical guidelines of the 1975 Declaration of Helsinki (and following revisions). All patients submitted to liver resection for HCC were enrolled in 1 year. Exclusion criteria included: the execution of local ablation therapies prior to surgery, final histological diagnosis other than HCC, presence of positive surgical margins (R1), and age < 18 years. Collected clinical data included (see also Table 1): patients’ age and gender, presence of cirrhosis, etiology of liver disease (when known), α-fetoprotein (ng/mL) before surgery, days of hospitalization, days of intensive care unit (ICU) stay, kind of surgery performed (wedge *versus* segmentectomy, laparotomy *versus* laparoscopy), clamping minutes (if performed) and transfusion. At least 1 year of follow-up was required; local or distant tumor recurrence was chosen as event, and disease-free survival (DFS) as the study end-point. No additional systemic or local therapies were performed after surgical resection.

**Table 1:** Clinical and pathological variables of the 67 cases

Variable	Result
Male gender (*n*. %)	51 (76.1%)
Age (mean ± SD)	67.8 ± 9.8 years
Cirrhosis (*n*. %)	30 (44.8%)
HCV/HBV infection (*n*. %)	31 (46.3%)
NASH (n. %)	14 (20.9%)
Hospital stay (mean ± SD)	14.7 ± 10.5 days
ICU stay (mean ± SD)	1.6 ± 1.5 days
Laparotomy (*n*. %)	62 (92.5%)
Blood transfusion (*n*. %)	8 (11.9%)
Tumor dimensions (mean ± SD)	5.4 ± 4.2 cm
Edmondson’s grade 3 or 4 (*n*. %)	51 (76.1%)
Macrotrabecular and/or solid architecture (*n*. %)	35 (52.2%)
Infiltrative margins (*n*. %)	34 (50.7%)
MVI (*n*. %)	55 (82.1%)
MPVI (*n*. %)	31 (46.3%)

*ICU *intensive care unit; *MVI* microvascular invasion; *MPVI *microscopic portal vein invasion; *SD* standard deviation

### Histopathological variables

Surgical specimens from liver resections were sent to the Pathology Unit, where they were sampled according to the routine protocols. Samples were fixed in formalin, embedded in paraffin (FFPE), and routinely processed. For each case, one representative FFPE block was chosen, and sections were cut for the evaluation of the histopathological variables at Haematoxylin–Eosin and for DNA extraction for next-generation sequencing (NGS).

The following histopathological data were collected (Table 1):

- Tumor dimensions, assessed during the macroscopical examination (in cm).
- Tumor grade according to Edmondson and Steiner [16] (Grade from 1 to 4) and WHO [17] (good, moderate, and poor differentiation).
- Tumor architecture, assessed as microtrabecular-acinar, macrotrabecular, or solid: for statistical purposes, and according to our previous data, architecture was also divided in “good architecture”, i.e. microtrabecular and acinar, and “bad architecture”, i.e. when areas of macrotrabecular and/or solid architecture were seen. According to the WHO guidelines, no specific cut-offs for the extension of “bad” areas were applied [17].
- Presence of infiltrative margins.
- Presence of overall microvascular invasion (MVI), and—separately—microscopical portal vascular invasion (MPVI) [5].
- Association with cirrhosis.

### Next-generation sequencing

DNA from FFPE specimens was extracted starting from 2 to 4 subsequent sections of 10 μm, using the QuickExtract FFPE DNA extraction kit (LGC Biosearch Technologies, Berlin, Germany), taking into account the areas of interest identified on the control slide stained with haematoxylin and eosin (H&E) by a pathologist.

DNA was quantified using Qubit dsDNA BR assay kit (Thermo Fisher Scientific, Waltham, MA, USA). NGS was performed using a multi-gene panel that allows the amplification of a total of 330 amplicons (human reference sequence hg19/GRCh37, 21.77 kb), including relevant alterations in *CTNNB1*, *TP53*, and *TERT* promoter, as previously described [15, 18]. Sequencing was performed using the Gene Studio S5Primemachine (Thermo Fisher Scientific, Waltham, MA, USA), and raw data analysis was conducted using the IonReporter tool (version 5.18, Thermo Fisher Scientific) and GoldenHelix GenomeBrowse (https://www.goldenhelix.com/products/GenomeBrowse/index.html).

According to the previously reported publication [19], only variants present in at least 5% of the total number of reads were considered. The Varsome tool (https://varsome.com/, accessed in April 2023) [20] was used to evaluate the pathogenicity of each variant.

### Statistical analysis

Statistical analysis was performed by means of SPSS® software for Windows, ver. 20. Data are reported as means ± standard deviations, frequencies, and percentages. The chi-square test was used to correlate variables. Multivariate Cox regression analysis was used to assess disease-free survival, using all the clinical and pathological variables listed in the “Materials and methods” section, as well as in Table 1. Univariate analyses were carried out by means of log-rank analysis and the Kaplan–Meier curve. A receiver operator characteristics (ROC) curve was built to assess the best cut-off value for tumor dimensions. A *p*-value ≤ 0.05 was used to exclude the null hypothesis.

## Results

### Clinical data and follow-up

Sixty-seven patients satisfied the inclusion criteria, 51 (76.1%) males and 16 (23.9%) females, with a mean age at surgery of 67.8 ± 9.8 years (range 43–85 years). The baseline clinical-pathological characteristics are summarized in Table 1. Thirty (44.8%) patients were cirrhotic; a history of HCV infection was recorded in 23 (34.3%), HBV infection in 8 (11.9%), and NASH in 14 (20.9%). Mean α-fetoprotein before surgery was 7322.09 ng/mL, with a very wide range (1.6–250,753 ng/mL); mean hospital stay was 14.7 ± 10.5 days, mean ICU stay was 1.6 ± 1.5 days. Wedge resection was performed in 60 (89.6%) cases, by laparotomy in 62 (92.5%); mean clamping time was 21.7 ± 31.2 min (range 0–193 min), transfusions were needed in 8 (11.9%) cases.

After a mean follow-up of 445 ± 481 days, 13 (19.4%) patients experienced HCC recurrence, intrahepatic in 9 cases, extrahepatic in 2 cases (one to the lung, one to abdominal lymph nodes), and both in 2; the mean recurrence time was 240 ± 249 days (range 42–900 days).

### Histopathological data and mutations

At histopathological analysis, the mean tumor dimension was 5.4 ± 4.2 cm (range 1–17 cm). Only one HCC (1.5%) was grade 1 according to Edmondson (i.e. well-differentiated/G1 according to WHO), 15 (22.4%) were grade 2, 32 (47.8%) were grade 3, and 19 (28.4%) were grade 4 (i.e. poorly differentiated/G3 according to WHO).

As far as tumor architecture is concerned, 32 (47.8%) cases showed a “good architecture”, i.e. microtrabecular and/or acinar, while 35 (52.2%) showed “bad architecture”, defined as areas of macrotrabecular or solid architecture; 4 of these cases were of the macrotrabecular massive subtype [21]. Infiltrative margins were observed in 34 (50.7%) cases.

MVI was present in 55 (82.1%) cases: 24 was of the “conventional” capillary type, while 31 (46.3% of the total) were classified as MPVI.

### Next-generation sequencing

The most frequent mutations in our series were *TERT* promoter (*n* = 41, 61.2%), *TP53* (*n* = 18, 26.9%), and *CTNNB1* (*n* = 17, 25.4%), with incidences comparable with what was reported in the literature. Concomitant mutations of *TERT* promoter and *TP53* were observed in 11 (16.4%) cases, concomitant mutations of *TERT* and *CTNNB1* in 13 (19.4%).

The incidences of the most frequent mutations of the three genes are summarized in detail in Table 2. *TP53* mutations characterized the HCC with worse morphological characteristics since it correlated with macrotrabecular/solid architecture (p = 0.043, chi-square test) and high Edmondson’s grade (*p* = 0.046). No correlations were found among *TERT* and *CTNNB1* mutations and histopathology (*data not shown*), but among the clinical variables HCV infection correlated with *TERT* mutation: 18 out of 23 (78.3%) HCV-positive cases were mutated (*p* = 0.033, chi-square test, see also Supplementary Table 1).

**Table 2:** Description and frequency of the single mutation of the *TERT*, *TP53* and *CTNBB1* genes found in our 67 cases

Gene	Mutation (number of cases)
*TERT* (*n* = 41)	c.-124C > T (*n* = 38), c.-146C > T (*n* = 3)
*TP53* (*n* = 18)	p.Arg158Cys (n = 1), p.Gln167Ter (*n* = 1), p.Gln192Lys (*n* = 2), p.Leu194SerfsTer15 (*n* = 1), p.Tyr205Cys (*n* = 3), p.Arg209Ter (*n* = 1),, p.Tyr220Cys (*n* = 1), p.Gly226Cys (n = 1), p.Asn239LysfsTer25 (*n* = 1), p.Ser240ThrfsTer6 (*n* = 1), p.Gly245Val (*n* = 1), p.Met246Val (*n* = 1), p.Arg273Leu (*n* = 1), p.Cys275Arg (*n* = 1), p.Lys320Ter (*n* = 1)
*CTNNB1* (*n* = 17)	p.Asp32Ala (n = 2), p.Asp32Gly (*n* = 1), p.Asp32Tyr (*n* = 1), p.Asp32Val (*n* = 2), p.Ser33Ala (n = 1), p.Gly34Glu (*n* = 1), p.Ile35Ser (*n* = 1), p.His36Pro (*n* = 1), p.Ser37Phe (*n* = 1), p.Thr41Ala (*n* = 1), p.Ser45Ala (*n* = 1), p.Ser45del (*n* = 1), p.Ser45Pro (n = 1), p.Ser45Tyr (*n* = 1), p.Ser348Tyr (*n* = 1)

### A combined histopathological-genetic algorithm predictive of HCC recurrence after resection

The influence of clinical variables towards DFS was analyzed to avoid biases: the clinical variable predictive of HCC recurrence was serum α-fetoprotein (p = 0.003, log-rank analysis), as already described in the literature [22]. At any chance, no correlations were found between the clinical variables (including α-fetoprotein) and the molecular biology results. The correlations among the main study variables are summarized in Supplementary Table 1.

**Fig. 1:**
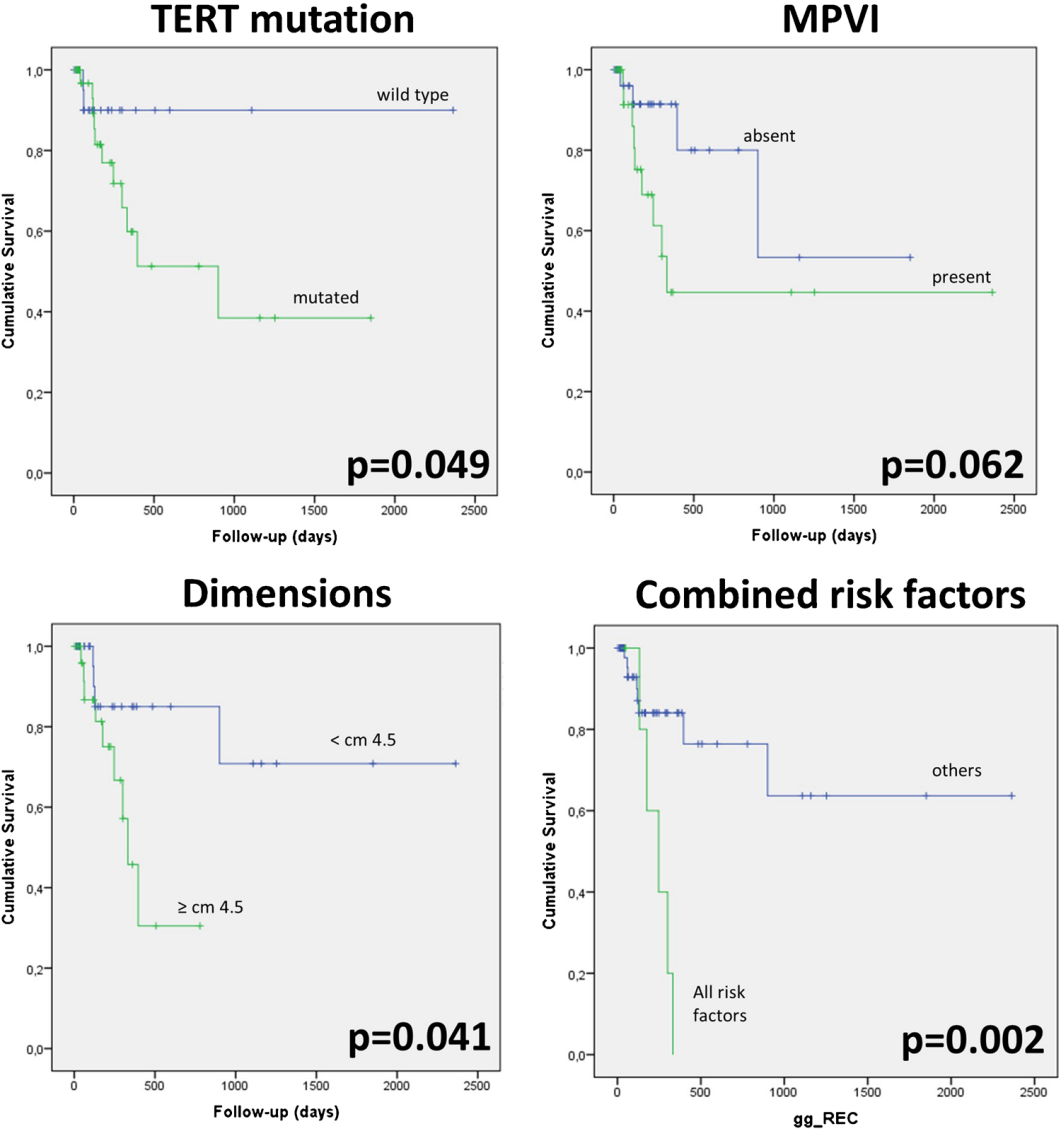
Kaplan–Meier curves of disease-free survival according to the three analyzed tissue variables, and their combinations

Among the histopathological and molecular variables, three correlated with disease-free survival at univariate analysis (Fig. 1): *TERT* promoter mutation, MPVI, and tumor dimensions. In particular, 11 out of 41 (26.8%) *TERT*-mutated HCC experienced recurrence, *versus* 2 out of 26 (7.7%) not mutated cases (*p* = 0.049; OR = 4.4; 95%CI 0.9–21.8). Nine out of 31 (29.0%) HCC with MPVI experienced recurrence, versus 4 out of 36 (11.1%) HCC without MPVI (*p* = 0.062; OR = 3.3; 95%CI 0.9–11.9). Concerning tumor dimensions, the ROC curve built to assess the sensitivity and specificity towards HCC recurrence showed an AUC of 0.675: taking 4.5 cm as the cut-off value (which is very close to the 5-cm defining the stage pT3) [23], we found 69% sensitivity and 62% specificity. In particular, 9 out of the 29 (31.0%) HCC ≥ cm 4.5 in dimensions experienced recurrence, versus 4 out of 37 HCC < 4.5 cm (*p* = 0.041; OR = 3.7; 95%CI 1.0–13.7).

The univariate analyses and the survival curves concerning the other pathological and genetic variables are represented in Supplementary Fig. 1.

**Fig. 2:**
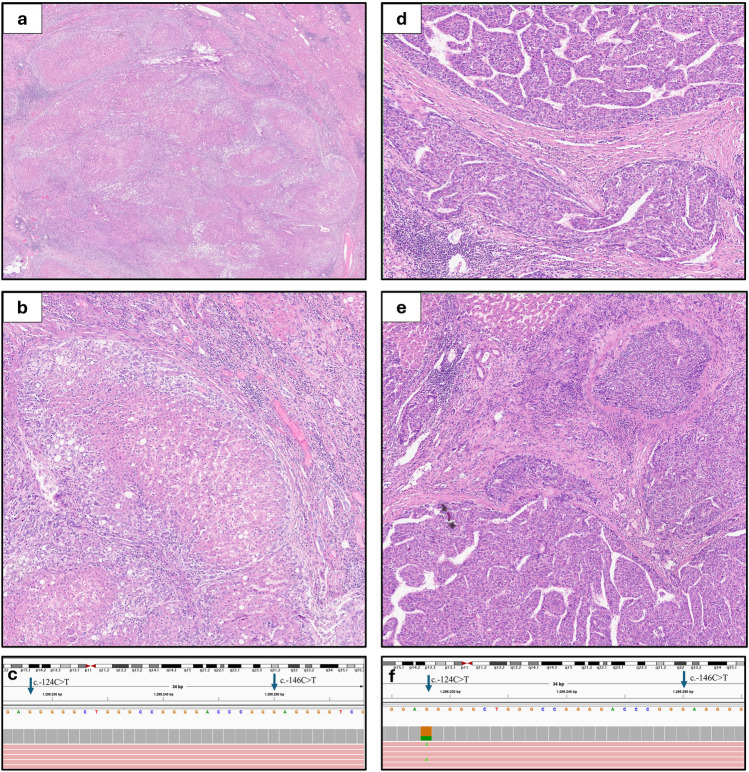
On the left, a not recurrent case from our series: (**a**, **b**) at Haematoxylin–Eosin stain, it is an Edmondson’s grade 2 HCC, with microtrabecular and acinar architecture, no MPVI and (**c**) wild-type *TERT*. On the right, a recurrent case: (**d**, **e**) a macrotrabecular-massive Edmondson’s grade 3 HCC, with MPVI and (**f**) characterized by the *TERT* c.-124C > T mutation

The multivariate Cox regression analysis confirmed the correlation of the three variables with DFS (Fig. 2):

- Tumor dimensions (*p* = 0.040, log-rank analysis; Exp(B) 1.13)
- MPVI (*p* = 0.010; Exp(B) 36.29)
- *TERT* promoter mutation (*p* = 0.034; Exp(B) 6.95).

The combination of these variables tended to worsen the overall DFS, and the concomitant occurrence of these three variables, i.e. *TERT* mutation, MPVI, and dimension more than 4.5 cm was present in 7 cases, among which 5 recurrence cases were recorded (*p* = 0.002; OR = 15.94; 95%CI 2.6–96.5), all in a relatively short time (Fig. 1).

## Discussion

**Fig. 3:**
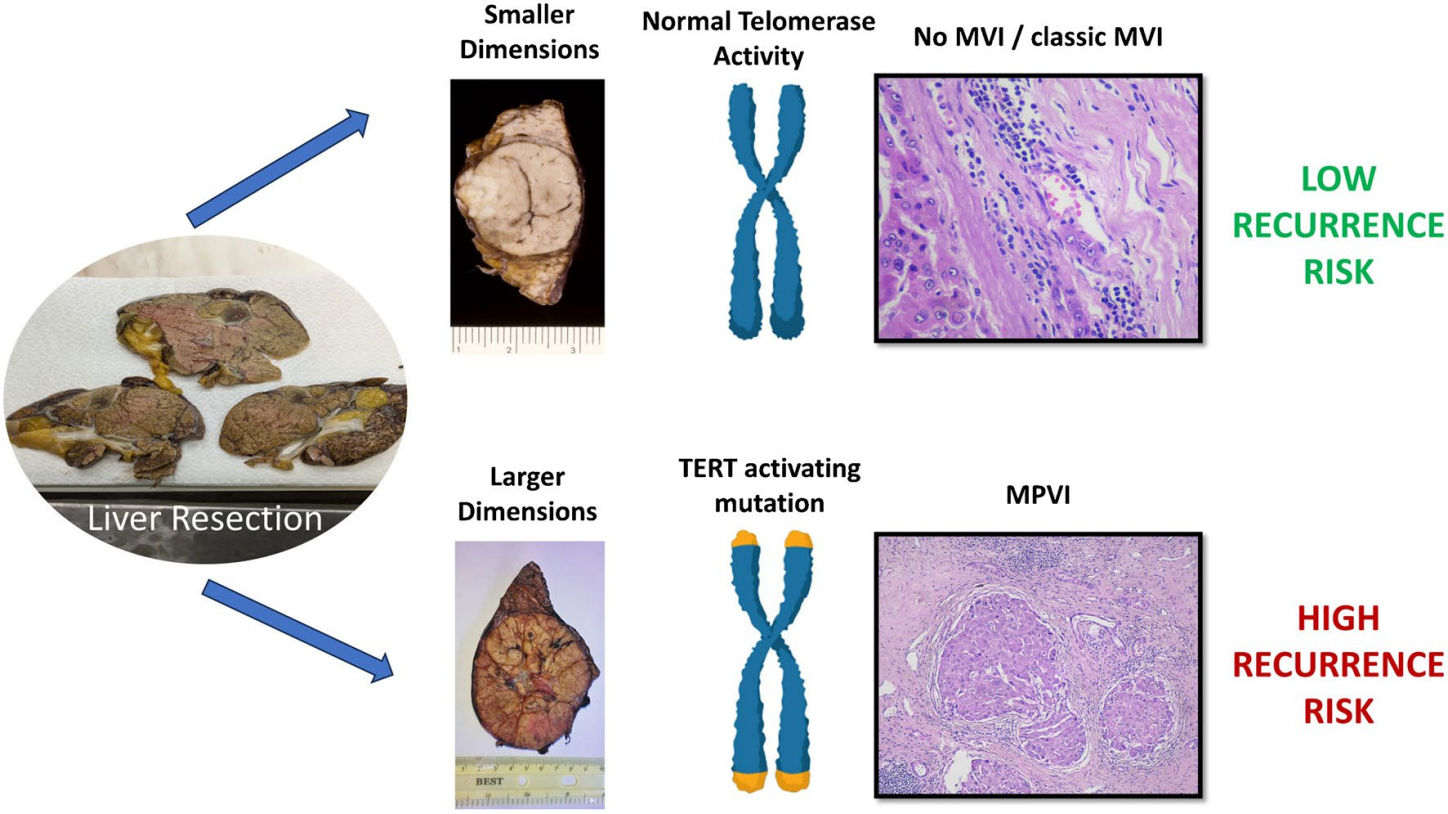
Graphical representation of the results, schematizing the three main tissue variables for the definition of a “low risk” versus “high risk” of hepatocellular carcinoma recurrence after liver resection

In today’s clinical practice, the pathologist can provide limited information to the surgical team after liver resection for HCC, especially concerning the prediction of DFS. The occurrence of MVI is still considered the most important prognosis predictor [3, 4], while recently the new WHO classification identified some HCC subtypes with worse prognosis, in particular, the massive macrotrabecular subtype, but providing few indications on the specific risk [17]. The application of molecular biology in HCC is still limited to research, with some attempts to reach a “molecular classification” for HCC [10, 13]. Our results showed that the synergic use of histopathological variables and molecular biology, feasible in most tertiary referral centers nowadays, can boost the prognostic value of post-surgical HCC analysis. Particularly, three variables—tumor dimensions, MPVI, and *TERT* mutation—were shown to be strongly predictive of post-surgical tumor recurrence in our prospective cohort of patients, on both multivariate and univariate analyses, and the combinations among these variables were even stronger (Fig. 3). To our knowledge, this is the first description of the synergic role of these histological and molecular features in resected HCC. Albeit none of these three variables should be a surprise, since they were already described to play a role in HCC biological behavior, they were always studied as independent risk factors, not combined. Other “classic” histological risk factors, such as Edmondson’s grade, architecture, margins, and cirrhosis showed different survival curves, but without reaching statistical significance (Supplementary Fig. 1): a possible explanation is that our study focuses on resected HCCs. Indeed, in our institution, HCC surgical resection is the first therapeutic choice for large advanced tumors, with compensated liver function. This might explain the relatively high prevalence of risk factors in our cohort.

The tumor dimension variable is not a surprise at all, since it is the main driver of local staging systems and therefore of prognosis: in the recent literature, HCC dimensions > 3 cm were described to be an independent risk factor for tumor recurrence after 1 year from the resection, on a large cohort of cases [23]. On the other hand, the cut-off dimension of 5 cm represents the passage from pathological stage 2 to stage 3 in HCCs according to the American Joint Committee on Cancer (AJCC) [24], as well as the criteria for transplantability according to the original Milan’s criteria [25]. The 4.5-cm cut-off that emerged from our series (very close to 5 cm) strongly confirms the literature findings, in addition to indicating the lack of biases in our cohort.

MVI is a well-known prognostic factor for HCC, as it is for many other malignancies [22]: the reported incidence in resected HCC is very variable, according to the different populations, and surgical settings, and considering the sampling biases during pathological examination as well [2, 3]. The tumor invasion of the portal veins—MPVI—was recently described to be a more significant prognostic factor than the “simple” pericapsular MVI [5]: this finding is of utmost importance, not only for the discovery per se but also because MPVI is a much more reproducible histopathological feature than MVI alone, with a better interobserver and intraobserver agreement. Of note, our multivariate and univariate analyses found out MPVI as one of the three predictors of HCC recurrence after resection.

The mutation for the *TERT* promoter region represents the last prognostic feature found in our cohort. *TERT* mutations are very frequent in HCC [10, 11], as confirmed by our series which showed more than 60% mutated cases, but the clinical implications are still unclear. The shortening of the telomere length was observed in chronic liver damage and cirrhosis, with the same mechanisms described for senescence [26]. The accumulation of genetic alterations during chronic damage leads to *TERT* mutation, with its reactivation and the immortalization of the cell, which is a very early event in hepatocarcinogenesis [27]. Some studies correlated *TERT* mutation with poorer prognosis in advanced HCC, but notably, these cohorts showed exclusively HBV and/or HCV-induced chronic liver disease [11, 14]. This is partly confirmed by our finding that 78% of HCV-related cases had *TERT* mutation. However, more than half of *TERT*-mutated cases had no history of viral infection, proving that viral infection correlates with a higher incidence of *TERT* mutations, but also that this association is not exclusive (see also Supplementary Table 1).

The main limitation of this study is due to the sample size. While it is true that the cohort of samples can be increased in future studies, it should be considered that our results showed that the predictive power of the recurrence event increases from 61% using “traditional” predictive markers (i.e. dimensions and MPVI), to 85% using *TERT* mutations. With this post hoc evaluation, we calculated a study power of 95%.

In conclusion, none of the described prognostic parameters should represent a novelty for surgeons and clinicians, but our results show that NGS analysis in resected HCC could not only be used for future therapeutic options but should be integrated with histopathological analysis in order to predict the risk of tumor recurrence after surgical resection. Indeed, in resected patients, *TERT* mutation is among the strongest predictors of tumor recurrence, together with tumor dimensions (i.e. pathological stage) and the occurrence of portal microvascular invasion, which should always be described separately from the classic MVI in the histopathological report.

## Supplementary information

Below is the link to the electronic supplementary material.

Supplementary information Suppl. Figure 1. Kaplan-Meier curves of disease-free survival according to the clinical, pathological, and molecular variables which did not enter the multivariate analysis. Other variables (such as tumor grade, architecture, and TP53 mutations) show different curves, but without statistical significance (TIF 2374 KB)

Supplementary file2 (DOCX 20 KB)

## Data Availability

Data available within the article or its supplementary materials.

## References

[1] European Association for the Study of the Liver (2018) EASL clinical practice guidelines: management of hepatocellular carcinoma. J Hepatol 69:182–236 29628281 10.1016/j.jhep.2018.03.019

[2] Nevola R, Ruocco R, Criscuolo L et al (2023) Predictors of early and late hepatocellular carcinoma recurrence. World J Gastroenterol 29:1243–1260 36925456 10.3748/wjg.v29.i8.1243 PMC10011963

[3] Imamura H, Matsuyama Y, Tanaka E et al (2003) Risk factors contributing to early and late phase intrahepatic recurrence of hepatocellular carcinoma after hepatectomy. J Hepatol 38:200–207 12547409 10.1016/s0168-8278(02)00360-4

[4] Zhang X, Li J, Shen F et al (2018) Significance of presence of microvascular invasion in specimens obtained after surgical treatment of hepatocellular carcinoma. J Gastroenterol Hepatol 33:347–354 28589639 10.1111/jgh.13843

[5] Kang I, Jang M, Lee JG et al (2021) Subclassification of microscopic vascular invasion in hepatocellular carcinoma. Ann Surg 274:e1170–e1178 31972640 10.1097/SLA.0000000000003781

[6] Vasuri F, Fittipaldi S, De Pace V et al (2018) Tissue miRNA 483–3p expression predicts tumor recurrence after surgical resection in histologically advanced hepatocellular carcinomas. Oncotarget 9:17895–17905 29707155 10.18632/oncotarget.24860 PMC5915163

[7] Vasuri F, Visani M, Acquaviva G et al (2018) Role of microRNAs in the main molecular pathways of hepatocellular carcinoma. World J Gastroenterol 24:2647–2660 29991871 10.3748/wjg.v24.i25.2647 PMC6034147

[8] Ng PKS, Lau CPY, Lam EKY et al (2018) Hypermethylation of NF-κB-activating protein-like (NKAPL) promoter in hepatocellular carcinoma suppresses its expression and predicts a poor prognosis. Dig Dis Sci 63:676–686 29353445 10.1007/s10620-018-4929-3

[9] Yu MC, Lee CW, Lin CH et al (2020) Differential hypermethylation of the VTRNA2-1 promoter in hepatocellular carcinoma as a prognostic factor: Tumor marker prognostic study. Int J Surg 79:282–289 32417463 10.1016/j.ijsu.2020.05.016

[10] Maloberti T, De Leo A, Sanza V et al (2022) Correlation of molecular alterations with pathological features in hepatocellular carcinoma: literature review and experience of an Italian center. World J Gastroenterol 28:2854–2866 35978866 10.3748/wjg.v28.i25.2854 PMC9280731

[11] Pezzuto F, Izzo F, De Luca P et al (2021) Clinical significance of telomerase reverse-transcriptase promoter mutations in hepatocellular carcinoma. Cancers (Basel) 13:3771 34359670 10.3390/cancers13153771 PMC8345216

[12] Lee JS (2015) The mutational landscape of hepatocellular carcinoma. Clin Mol Hepatol 21:220–229 26523267 10.3350/cmh.2015.21.3.220 PMC4612282

[13] Calderaro J, Couchy G, Imbeaud S et al (2017) Histological subtypes of hepatocellular carcinoma are related to gene mutations and molecular tumour classification. J Hepatol 67:727–738 28532995 10.1016/j.jhep.2017.05.014

[14] Oh BK, Kim H, Park YN et al (2008) High telomerase activity and long telomeres in advanced hepatocellular carcinomas with poor prognosis. Lab Invest 88:144–152 18158557 10.1038/labinvest.3700710

[15] Chillotti S, Maloberti T, Degiovanni A et al (2023) Hepatocellular carcinomas with concomitant mutations of TERT, TP53, and CTNNB1: is there a role for artificial intelligence? Crit Rev Oncog 28:31–35 37968991 10.1615/CritRevOncog.2023049650

[16] Edmondson HA, Steiner PE (1954) Primary carcinoma of the liver: a study of 100 cases among 48,900 necropsies. Cancer 7:462–503 13160935 10.1002/1097-0142(195405)7:3<462::aid-cncr2820070308>3.0.co;2-e

[17] WHO Classification of Tumours - Digestive System Tumours, 5th edn. IARC: Lyon

[18] Malvi D, Vasuri F, Maloberti T et al (2022) Characterization of pancreatic ductal adenocarcinoma using a next-generationsequencing custom-designed multigene panel. Diagnostics (Basel) 12:1058 35626213 10.3390/diagnostics12051058 PMC9139796

[19] De Biase D, Acquaviva G, Visani M et al (2020) Molecular diagnostic of solid tumor using a next generation sequencing custom-designed multi-gene panel. Diagnostics 10:250 32340363 10.3390/diagnostics10040250 PMC7236002

[20] Kopanos C, Tsiolkas V, Kouris A et al (2019) VarSome: the human genomic variant search engine. Bioinformatics 35:1978–1980 30376034 10.1093/bioinformatics/bty897 PMC6546127

[21] Vasuri F, Fittipaldi S, Giunchi F et al (2016) Facing the enigma of the vascular network in hepatocellular carcinomas in cirrhotic and non-cirrhotic livers. J Clin Pathol 69:102–108 26243063 10.1136/jclinpath-2015-203028

[22] Costentin C, Audureau E, Park YN, et al (2023) ERS: a simple scoring system to predict early recurrence after surgical resection for hepatocellular carcinoma. Liver Int. 10.1111/liv.15683 10.1111/liv.15683 37577984

[23] Amin MB, Edge SB, Greene FL et al (2017) American Joint Committee on Cancer Manual (AJCC), 8th ed. New York: Springer

[24] Jung SM, Kim JM, Choi GS et al (2019) Characteristics of early recurrence after curative liver resection for solitary hepatocellular carcinoma. J Gastrointest Surg 23:304–311 30215196 10.1007/s11605-018-3927-2

[25] Mazzaferro V, Regalia E, Doci R et al (1996) Liver transplantation for the treatment of small hepatocellular carcinomas in patients with cirrhosis. N Engl J Med 334:693–699 8594428 10.1056/NEJM199603143341104

[26] Kitada T, Seki S, Kawakita N et al (1995) Telomere shortening in chronic liver diseases. Biochem Biophys Res Commun 211:33–39 7779103 10.1006/bbrc.1995.1774

[27] Quaas A, Oldopp T, Tharun L et al (2014) Frequency of TERT promoter mutations in primary tumors of the liver. Virchows Arch 465:673–677 25267585 10.1007/s00428-014-1658-7

