# Supplementary figures and images for: Beyond histology: A tissue algorithm predictive of post-surgical recurrence in hepatocellular carcinomas, including TERT promoter mutation
Source: Virchows Arch. 2024 May 18;486(2):365–72. doi: 10.1007/s00428-024-03791-y (PMC11876287; doi:10.1007/s00428-024-03791-y)

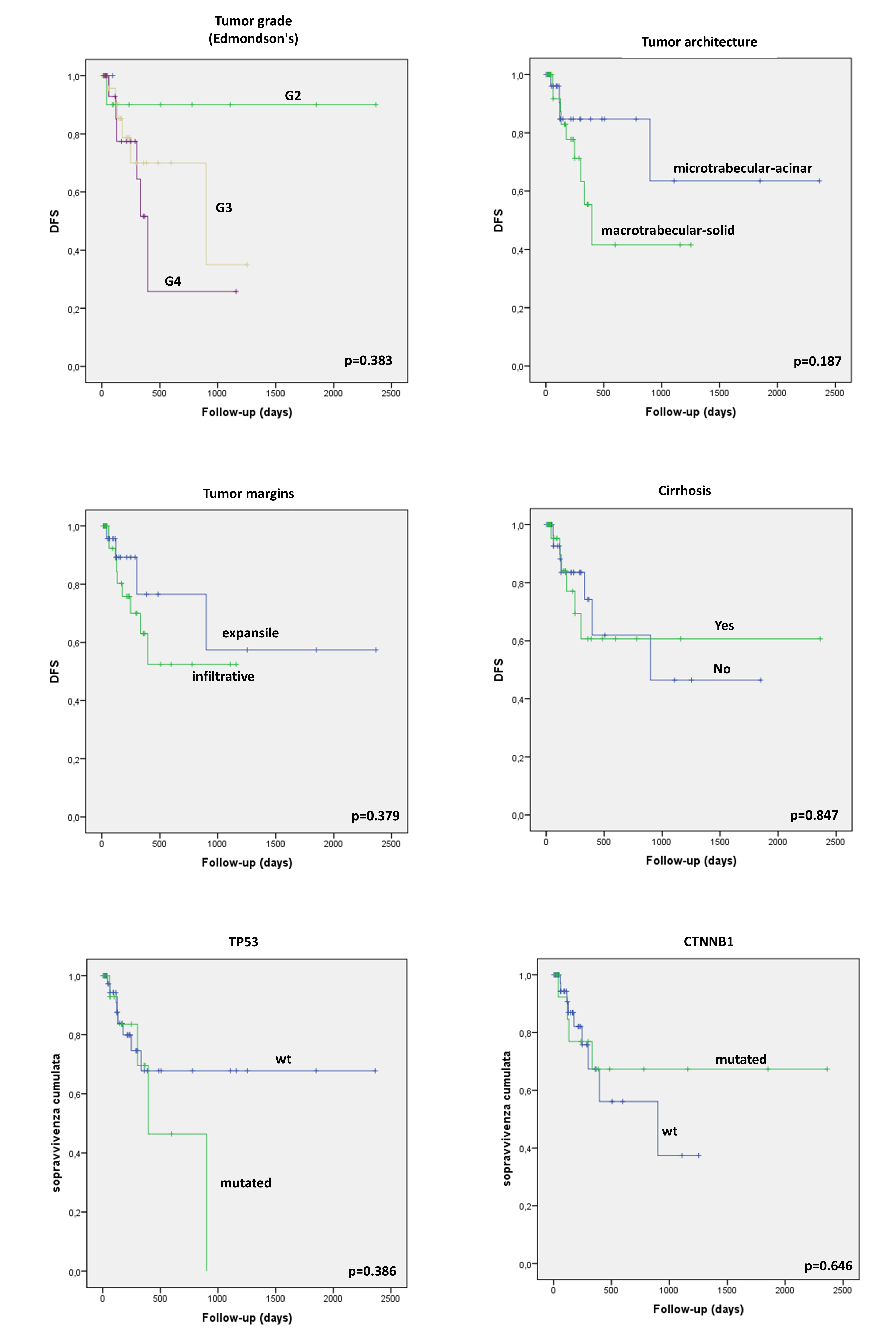

Supplement: Supplementary file 1 — Supplementary information Suppl. Figure 1. Kaplan-Meier curves of disease-free survival according to the clinical, pathological, and molecular variables which did not enter the multivariate analysis. Other variables (such as tumor grade, architecture, and TP53 mutations) show different curves, but without statistical significance (TIF 2374 KB) [file 428_2024_3791_MOESM1_ESM.tif]
