# Supplementary material for: Beyond histology: A tissue algorithm predictive of post-surgical recurrence in hepatocellular carcinomas, including TERT promoter mutation
Source: Virchows Arch. 2024 May 18;486(2):365–72. doi: 10.1007/s00428-024-03791-y (PMC11876287; doi:10.1007/s00428-024-03791-y)
Supplement: Supplementary file 2 — Supplementary file2 (DOCX 20 KB) [file 428_2024_3791_MOESM2_ESM.docx]

|  | | **TERT** | | **MPVI** | | **dimensions** | | **Edmondson's grade** | | **architecture** | | **margins** | | **HCV infection** | | **cirrhosis** | |
| --- | --- | --- | --- | --- | --- | --- | --- | --- | --- | --- | --- | --- | --- | --- | --- | --- | --- |
|  |  | **wt** | **mutated** | **absent** | **present** | **<4.5** | **≥4.5** | **1-2** | **3-4** | **"good** | **"bad"** | **expansile** | **infiltrative** | **absent** | **present** | **absent** | **present** |
| **TERT** | wt |  |  | 14 | 12 | 14 | 12 | 9 | 17 | 15 | 11 | 15 | 10 | 21 | 5 | 18 | 8 |
|  | mutated |  |  | 22 | 19 | 23 | 17 | 7 | 34 | 17 | 24 | 16 | 24 | 23 | 18 | 18 | 22 |
| total | |  |  | 36 | 31 | 37 | 29 | 16 | 51 | 32 | 35 | 31 | 34 | 44 | 23 | 36 | 30 |
| **sig.** |  |  |  | p=0.594 |  | p=0.484 |  | p=0.090 |  | p=0.148 |  | p=0.094 |  | **p=0.033** |  | **p=0.046** |  |
| **MPVI** | absent | 14 | 22 |  |  | 22 | 14 | 12 | 24 | 21 | 15 | 20 | 14 | 25 | 11 | 19 | 16 |
|  | present | 12 | 19 |  |  | 15 | 15 | 4 | 27 | 11 | 20 | 11 | 20 | 19 | 12 | 17 | 14 |
| total | | 26 | 41 |  |  | 37 | 29 | 16 | 51 | 32 | 35 | 31 | 34 | 44 | 23 | 36 | 30 |
| **sig.** |  | p=0.594 |  |  |  | p=0.256 |  | **p=0.046** |  | p=0.052 |  | p=0.051 |  | p=0.329 |  | p=0.580 |  |
| **dimensions** | <4.5 | 14 | 23 | 22 | 15 |  |  | 11 | 26 | 22 | 15 | 22 | 14 | 22 | 15 | 17 | 19 |
|  | ≥4.5 | 12 | 17 | 14 | 15 |  |  | 5 | 24 | 10 | 19 | 9 | 19 | 21 | 8 | 18 | 11 |
|  | total | 26 | 40 | 36 | 30 |  |  | 16 | 50 | 32 | 34 | 31 | 33 | 43 | 23 | 35 | 30 |
| **sig.** |  | p=0.484 |  | p=0.256 |  |  |  | p=0.189 |  | **p=0.038** |  | **p=0.020** |  | p=0.202 |  | p=0.173 |  |

**Supplemental Table 1.** Cross-correlations among the three variables each other and the other (most represented) clinico-pathological variables. The three variables correlated with HCC recurrence do not correlate each other.
